# The Cysteine-Containing Cell-Penetrating Peptide AP Enables Efficient Macromolecule Delivery to T Cells and Controls Autoimmune Encephalomyelitis

**DOI:** 10.3390/pharmaceutics13081134

**Published:** 2021-07-25

**Authors:** Won-Ju Kim, Gil-Ran Kim, Hyun-Jung Cho, Je-Min Choi

**Affiliations:** 1 Department of Life Science, College of Natural Sciences, Hanyang University, Seoul 04763, Korea; kwj8996@gmail.com (W.-J.K.); godqhr3079@gmail.com (G.-R.K.); chohj730@hanmail.net (H.-J.C.); 2 Research Institute for Natural Sciences, Hanyang University, Seoul 04763, Korea; 3 Research Institute for Convergence of Basic Sciences, Hanyang University, Seoul 04763, Korea

**Keywords:** T cell, immune regulation, cell-penetrating peptide, AP, CTLA-4, multiple sclerosis, experimental autoimmune encephalomyelitis EAE, drug delivery system

## Abstract

T cells are key immune cells involved in the pathogenesis of several diseases, rendering them important therapeutic targets. Although drug delivery to T cells is the subject of continuous research, it remains challenging to deliver drugs to primary T cells. Here, we used a peptide-based drug delivery system, AP, which was previously developed as a transdermal delivery peptide, to modulate T cell function. We first identified that AP-conjugated enhanced green fluorescent protein (EGFP) was efficiently delivered to non-phagocytic human T cells. We also confirmed that a nine-amino acid sequence with one cysteine residue was the optimal sequence for protein delivery to T cells. Next, we identified the biodistribution of AP-dTomato protein *in vivo* after systemic administration, and transduced it to various tissues, such as the spleen, liver, intestines, and even to the brain across the blood–brain barrier. Next, to confirm AP-based T cell regulation, we synthesized the AP-conjugated cytoplasmic domain of CTLA-4, AP-ctCTLA-4 peptide. AP-ctCTLA-4 reduced IL-17A expression under Th17 differentiation conditions *in vitro* and ameliorated experimental autoimmune encephalomyelitis, with decreased numbers of pathogenic IL-17A^+^GM-CSF^+^ CD4 T cells. These results collectively suggest the AP peptide can be used for the successful intracellular regulation of T cell function, especially in the CNS.

## 1. Introduction

The human immune system is indispensable for defending the body against various pathogens, maintaining health, and maintaining immune homeostasis [1]. As immune cells, various types of T cells circulate or reside in the body and play an important role in the adaptive immune system [2]. However, diseases, such as autoimmune diseases, can develop when the immune homeostasis is disrupted, and T cells attack the body by recognizing self-antigens [3]. Multiple sclerosis (MS) is a representative T cell-mediated autoimmune disease that is caused by inflammation of the central nervous system (CNS) by autoreactive myelin-specific T cells that cross the blood–brain barrier (BBB) [4].

T cell modulation is important for controlling diseases, such as allergic diseases [5], autoimmune diseases [6], and cancer [7]. Although many attempts have been made to modulate the function of T cells, most of these approaches have been limited to extracellular targets because of the characteristics of antibodies [8,9]. Although there are numerous molecules inside the cellular environment which could serve as potential targets [10,11,12], only small-molecule drugs are available to effectively target intracellular molecules [13].

Cell-penetrating peptides are commonly composed of 5–30 amino acid residues and are capable of carrying cargo molecules, such as peptides, proteins, oligonucleotides and small molecules, into cells [14]. For two decades, since the function of the TAT protein of HIV was characterized in 1988 [15,16], numerous studies have been conducted to develop CPP-based drugs, and there are eight candidates in clinical trials with optimistic results for FDA approval [17]. Recently, the range of diseases that can be targeted has increased with the identification of novel CPPs that penetrate biological tissue barriers. The blood–brain barrier (BBB)-permeable peptide, dNP2, can deliver a T cell modulatory protein (ctCTLA-4) to the central nervous system (CNS) and ameliorate autoimmune encephalomyelitis [18,19]. A novel transdermal delivery peptide, AP, enabled the transcutaneous delivery of phosphatase and alleviated skin inflammation [20]. In a previous study, we described the development of a novel nine-amino acid transdermal delivery peptide, AP, derived from the human astrotactin-1 protein. AP showed significant delivery efficiency in both mouse and human skin tissue [20]. The characteristics of transcutaneous delivery were confirmed; however, the delivery efficiency in T cells and their ability to cross the BBB while carrying cargo remain unclear.

In this study, we identified that AP exhibited potent delivery efficiency to primary human T cells and Jurkat T cells carrying the EGFP protein. Cysteine is a key amino acid that enhances delivery efficiency. In systemic administration, AP-dTomato showed significant tissue distribution, especially in the spleen, lung, and even brain tissue, as visualized *in vivo* using live multiphoton confocal microscopy. We synthesized the AP-conjugated cytoplasmic domain of CTLA-4, AP-ctCTLA-4, which showed significant suppression of IL-17A and induction of Foxp3 *in vitro* as well as the alleviation of experimental autoimmune encephalomyelitis (EAE), with reduced levels of pathogenic IL-17A^+^GM-CSF^+^ CD4 T cells.

## 2. Materials and Methods

### 2.1. DNA Cloning 

CPP-EGFP, including AP-EGFP, was synthesized by Cosmo Genetech (Seoul, Korea), and the target genes were amplified by PCR using the Pfu Plus PCR Master Mix (Elpis Biotech, Daejeon, Korea). The amplified sequences for CPP-EGFP were ligated with inserted restriction sites NheI and HindIII (New England Biolabs, Ipswich, MA, USA) and cloned into the pRSET-B protein expression vector using T4 DNA ligase (New England Biolabs). The ligated gene was transformed into DH5α *Escherichia coli* cells.

### 2.2. Purification of the Recombinant Proteins

The purification protocol was performed as previously described [20]. Recombinant fluorescent proteins conjugated to AP and control CPP peptides, were expressed and purified from *E. coli* BL21 (DE3). The cells were transformed with plasmids encoding recombinant fluorescent proteins. Protein expression was induced with 0.2 mM isopropyl β-D-1-thiogalactopyranoside (Gold Biotechnology, St. Louis, MO, USA), after which cells were cultured overnight at 20 °C with shaking at 150 rpm. The cells were then harvested by centrifugation at 5000 rpm for 10 min and resuspended in binding buffer (50 mM NaH_2_PO_4_, 300 mM NaCl, and 10 mM imidazole, pH 8.0). Cells were lysed using a Vibra-cell VCX-130 ultrasonic processor (Sonics and Materials Inc., Newtown, CT, USA). The lysate was clarified by centrifugation, and the supernatant was filtered through 0.45 μm cellulose nitrate syringe filters (Sartorius Stedim Biotech GmbH, Goettingen, Germany). The 6x His-tagged target proteins were purified via Ni-NTA affinity chromatography (Qiagen, Hilden, Germany). Finally, the purified proteins were desalted using a PD-10 Sephadex G-25 column with PBS containing 10% glycerol. Protein concentrations were measured using the Bradford assay, and the purified proteins were stored at 80 °C.

### 2.3. Cell Culture 

The Jurkat (human leukemia cells) cell line was purchased from ATCC (ATCC; Rockville, MD, USA) and cultured in Roswell Park Memorial Institute (RPMI) 1640 medium (Cellgro, Mediatech Inc., Herndon, VA, USA) containing 10% fetal bovine serum (FBS) (Cellgro) and 1% penicillin/streptomycin. All cells were cultured at 37 °C in a 5% CO_2_ incubator (Forma Scientific).

### 2.4. In Vitro T Cell Delivery 

Jurkat cells were seeded into 24-well plates (1 × 10^6^ cells per well) in RPMI medium. The cells were then treated with recombinant proteins at the indicated concentrations (0.5, 1, 2, or 5 μM) for 1 h or for different periods of time (10 min, 30 min, 1 h, 2 h, or 4 h) with 5 µM protein at 37 °C. Following incubation, the cells were washed three times with PBS to eliminate the uninternalized recombinant proteins on the cell membrane. Intracellular fluorescence was analyzed by flow cytometry (FACS Canto II, BD Bioscience), and the data were analyzed using FlowJo software (version 10.7.1; Tree Star, Inc., Ashland, OR, USA).

Primary hPBMCs were obtained from healthy donors by leukapheresis under a protocol approved by the Yale Human Investigations Committee. hPBMCs were seeded into 96-well plates (2 × 10^5^ cells per well) in RPMI medium. The cells were then treated with 10 µM of the recombinant proteins for 1 h at 37 °C. Following incubation, the cells were washed three times with PBS to eliminate uninternalized recombinant proteins on the cell membrane. Intracellular fluorescence was analyzed by flow cytometry (FACS Canto II, BD Bioscience), and the data were analyzed using FlowJo software (version 10.7.1; Tree Star, Inc.).

### 2.5. Intracellular Delivery Mechanisms 

Jurkat cells were seeded into 24-well plates (1 × 10^6^ cells per well) and preincubated with heparin (0, 10, 20, or 50 μg/mL) and MβCD (0, 3, or 5 mM) at 37 °C for 30 min. The cells were then treated with 5 μM recombinant protein for 1 h and washed as described above. The intracellular delivery efficiency was analyzed using flow cytometry.

Six- to eight-week-old mice for *in vitro* experiments, or eight-week-old female C57BL/6 mice for the EAE model, were purchased from Orient Bio. The weight of the mice was 19 ± 1.5 g in the *in vitro* experiments and 21 ± 1.5 g in the EAE mouse model. Mice were housed and bred in a specific pathogen-free animal facility at Hanyang University under controlled conditions with a constant temperature (21 ± 1 °C), humidity (50 ± 5%), and 12 h light/dark cycle and with regular chow and autoclaved water. All experimental procedures used in this study were approved by the Institutional Animal Care and Use Committee (IACUC) of Hanyang University (2020-0022A).

### 2.6. In Vivo Delivery 

Six- to eight-week-old C57BL/6 mice were intraperitoneally injected with 5 mg of AP-dTomato or control CPP-dTomato proteins to analyze the systemic delivery efficiency of AP-dTomato. The mice were euthanized 2 h after injection. The tissues were then harvested, washed with PBS, and fixed with 4% paraformaldehyde. All harvested tissues were frozen using OCT compound (Wako Chemical, Osaka, Japan). Cryosections were stained with Hoechst 33,342 dye for nuclei visualization and enveloped with Fluoroshield and DABCO mounting media. The slides were analyzed using a fluorescence microscope (Leica DMi8, Leica Microsystems, Wetzlar, Germany). Following acquisition, images were analyzed using ImageJ software (version 2.0.0; National Institutes of Health, Bethesda, MD, USA).

### 2.7. Multiphoton Microscopy of Live Animals 

The imaging protocol was performed as previously described [18]. For *in vivo* multiphoton imaging of the brain, male C57BL/6 mice (23–25 g) were subjected to surgery to introduce an observation window to the skull. Animals were anesthetized via isoflurane inhalation, and a body temperature of 37–38 °C was maintained using a homeothermic heating pad system controlled by a rectal probe. Isoflurane levels were set at 3% to induce anesthesia and were maintained at 1.5% during cranial window surgery or multiphoton imaging. The animals were closely monitored throughout the procedure to ensure their physiological health. All surgical procedures were approved by the IACUC of the Sungkyunkwan University. Animals were fixed in a stereotaxic frame (David Kopf Instruments, Los Angeles, CA, USA), and a round cranial window with a diameter of 3 mm was created on the right hemisphere, centered at ML (+2.5 mm) and AP (−1.5 mm). After craniotomy, a customized chamber plate (Narishige Inc., Tokyo, Japan) with a 5 mm observation hole was placed on the opened craniotomy area and fixed with dental resin. The cranial window was then filled with sterilized artificial cerebrospinal fluid (125 mM NaCl, 2.5 mM KCl, 25 mM NaHCO_3_, 1.25 mM NaH_2_PO_4_, 2 mM CaCl_2_, 1 mM MgSO_4_, 10 mM glucose, pH 7.4) and covered with a 7-mm coverslip. The cranial window was sealed with cyanoacrylic glue, and the animals were then placed in a head-fixing apparatus (MAG-1, Narishige Inc.) for multiphoton microscopy (TCS SP8 MP, Leica Microsystems CMS GmbH). Excitation was performed using a 900 nm Ti:sapphire laser (Chameleon Vision II, Coherent Inc., Santa Clara, CA, USA), and the emitted fluorescence signal was detected using a hybrid detector through a 585/40 band-pass filter cube. To track the delivery of the carrier peptide into the brain tissue, 3D z-stack images were obtained with a 20 min time interval for 2 h following the injection of the carrier peptide via the tail vein (2.5 mg/animal). The imaged brain size was 354.29 × 354.29 μm^2^ (1024 × 1024 pixels) and was acquired using a 25× water-immersion objective lens (N.A. 0.95). The imaging depth was approximately 450–500 μm from the brain surface, with a resolution of 1 μm. Following acquisition, the images were analyzed using LAS AF 3.2.0 (Leica Microsystems CMS GmbH) and Imaris 7.7.2 (Bitplane) software.

### 2.8. Peptide Synthesis

All peptides used in this study, including AP-ctCTLA-4 and dNP2-ctCTLA-4, were synthesized by solid-phase peptide synthesis. The purity of the final peptide product was >95% (AnyGen).

### 2.9. T Cell Differentiation

Naïve CD4 T cells were isolated from splenocytes of six- to eight-week-old C57BL/6 mice using a mouse naïve CD4+ T cell isolation kit (Miltenyi Biotec, Bergisch Gladbach, Germany) according to the manufacturer’s protocols. Purified naïve CD4 T cells were activated with plate-bound anti-CD3 (5 μg/mL; BD Pharmingen) and anti-CD28 (5 μg/mL; BD Pharmingen) antibodies, and differentiated with the following cytokine cocktails in a 96-well plate for four days: 5 µg/mL of anti-IFN-γ (eBioscience, San Diego, CA, USA), 5 µg/mL of anti-IL-4 (eBioscience), 2 ng/mL of recombinant murine TGF-β (R&D Systems, Minneapolis, MN, USA), 30 ng/mL of IL-6 (Peprotech, Rocky Hill, NJ, USA), 20 ng/mL of IL-23 (R&D Systems), 20 ng/mL of IL-1β (R&D Systems), and 50 U/mL of IL-2 (Peprotech) for Th17. The cells were cotreated with 2 µM of AP, AP-ctCTLA-4, or dNP2-ctCTLA-4 peptides and cytokine cocktails.

After incubation, cells were stained to exclude dead cells using the Zombie Aqua™ Fixable Viability Kit (BioLegend, San Diego, CA, USA) before antibody staining at room temperature for 10 min. After washing, the surface proteins of the cultured naïve CD4 T cells were stained with monoclonal antibodies for 15 min at 4 °C. To determine intracellular cytokines or transcription factor levels, cells were restimulated with the Cell Stimulation Cocktail (plus protein transport inhibitors) (Thermo Fisher, Waltham, MA, USA) for 4 h at 37 °C and stained with surface marker antibodies. Cells were then fixed, permeabilized using the Foxp3/Transcription Factor Staining Buffer Set (eBioscience), and stained with anti-mouse IL-17 and anti-mouse Foxp3. Cells were analyzed using a FACS Canto II flow cytometer and FlowJo software version 10.7.1.

### 2.10. EAE Induction 

The EAE induction protocol was performed as previously described [19]. Eight-week-old female C57BL/6 mice were purchased from Orient Bio. The protocol described in this study was approved by the Animal Experimentation Ethics Committee of Hanyang University. EAE was induced by subcutaneous immunization using the MOG35-55/CFA Emulsion PTX kit (Hooke Labs, Lawrence, MA, USA) according to the manufacturer’s protocol. Mice were anesthetized with isoflurane, and MOG35-55/CFA was subcutaneously injected bilaterally, followed by an intraperitoneal injection of pertussis toxin (PTX) 2 and 24 h later. The mice were randomized into different groups after MOG immunization. EAE scores were assessed daily using the following scoring system: 0, no obvious signs of disease; 0.5, partially limp tail; 1, completely limp tail; 1.5, limp tail and waddling gait; 2, paralysis of one hind limb; 2.5, paralysis of one hind limb and partial paralysis of the other hind limb; 3, paralysis of both hind limbs; 3.5, ascending paralysis; 4, paralysis of the trunk; 4.5, moribund; 5, death. AP-ctCTLA-4 or dNP2-ctCTLA-4 peptide (100 µg) was injected intraperitoneally. Mice were euthanized at the end of the experiments, and isolated spinal cords were digested with 1 mg/mL of DNase 1 (Sigma-Aldrich, St Louis, MO, USA ) and 1 mg/mL of collagenase D (Sigma-Aldrich) at 37 °C and incubated at 80 rpm in a shaking incubator (#BF-30SI, Biofree, Seoul, South Korea) for 40 min. After enzyme digestion, 500 mM EDTA was added, and lymphocytes were isolated by Percoll (GE Healthcare, Uppsala, Sweden) density-gradient centrifugation. Isolated infiltrated lymphocytes were restimulated with Cell Stimulation Cocktail (plus protein transport inhibitors) (Thermo Fisher, Waltham, MA, USA) for 4 h at 37 °C and stained with anti-mouse CD4. Cells were then fixed, permeabilized using the Foxp3/Transcription Factor Staining Buffer Set (eBioscience), and stained with anti-mouse IFN-γ, anti-mouse IL-17, anti-mouse GM-CSF, or anti-mouse Foxp3. Cells were analyzed using a FACS Canto flow cytometer and FlowJo software version 10.7.1.

### 2.11. Statistical Analysis

Data were analyzed by Mann–Whitney test, one-way ANOVA, or two-way repeated measure ANOVA using GraphPad Prism software (version 8.0; GraphPad, San Diego, CA, USA). Data are presented as the mean ± standard deviation or ±SEM. In all cases, significance was defined as *p* ≤ 0.05. The sample size and statistical analysis information are provided in the figure legends.

## 3. Results

### 3.1. Efficient Peptide-Based Protein Delivery System, AP to T Cells

**Figure 1:**
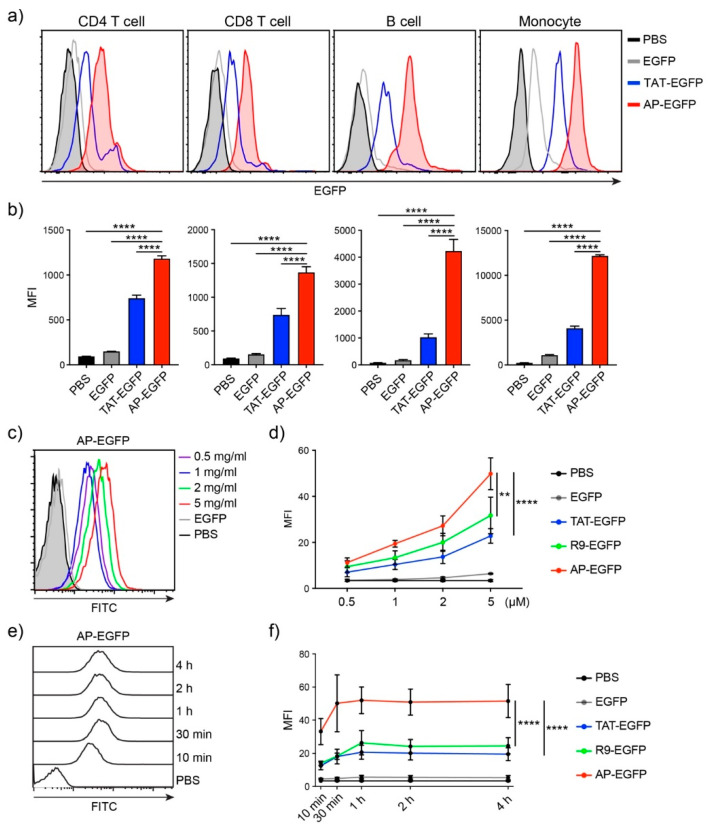
*In vitro* protein delivery efficiency into human T cells by the AP peptide (**a**) Comparison of delivery efficiencies to human PBMCs. Overall, 10 µM of each EGFP protein was treated for 1 h. Intracellular fluorescence was analyzed by flow cytometry. (**b**) The bar graph of mean fluorescent intensity (MFI). (**c**) Dose-dependent delivery efficiency of AP-EGFP. Each dose of AP-EGFP protein was delivered to Jurkat T cells for 1 h. (**d**) Comparison of delivery efficiency to Jurkat T cells. (**e**) Time-dependent delivery efficiency of AP-EGFP. Overall, 5 µM of AP-EGFP protein was delivered to Jurkat T cells for indicated periods. (**f**) Comparison of the delivery efficiency to Jurkat T cells. Error bars indicate SD ** *p* ≤ 0.01; **** *p* ≤ 0.0001.

In our previous study, we mainly focused on the delivery efficiency of AP to skin tissue and cells for the treatment of dermatitis [20], whether it is delivered to T cells or other organs by systemic administration has not been well described. First, to identify the delivery efficiency to T cells, which are difficult to deliver drugs to, we treated human peripheral blood mononuclear cells (hPBMCs) with AP-EGFP, which is mainly composed of B cells and T cells, and analyzed intracellular EGFP fluorescence by flow cytometry. The delivery efficiency of AP-EGFP was significantly higher than that of TAT-EGFP in monocytes, B cells, and even CD4 and CD8 T cells (Figure 1a,b). To further identify the characteristics of delivery efficiency in T cells, we treated Jurkat T cells with AP-EGFP in a dose- and time-dependent manner. Flow cytometric analysis demonstrated that AP-EGFP was delivered to Jurkat T cells in a dose-dependent manner (Figure 1c) and exhibited significantly higher delivery efficiency than TAT- and R9-EGFP, which are the most common CPPs (Figure 1d). Interestingly, AP-EGFP rapidly entered the cells within 10 min (Figure 1e), with a significantly higher intracellular fluorescence intensity than the controls used in the study (Figure 1f). These results suggest that AP could also be an effective CPP for delivering cargo into T cells.

### 3.2. T Cell Delivery Mechanism of the Optimized AP Peptide

**Figure 2:**
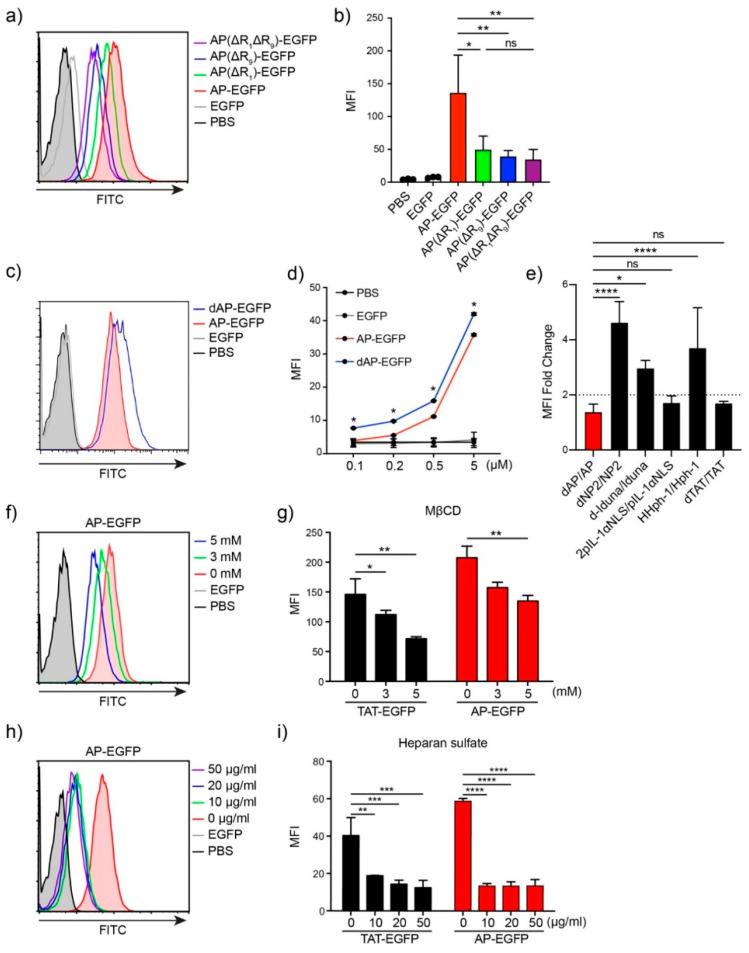
Optimization of the AP sequence and mechanism of protein delivery to T cells (**a**) Delivery efficiency of AP and its variants. Overall, 5 µM of each EGFP protein was treated for 1 h. Intracellular fluorescence was analyzed by flow cytometry. (**b**) The bar graph of mean fluorescent intensity (MFI). (**c**) Comparison of delivery efficiency of AP-EGFP and dAP-EGFP. Overall, 5 µM of each EGFP protein was treated for 1 h. (**d**) The bar graph of mean fluorescent intensity (MFI). (**e**) The fold change of MFI between the original form and tandem repeated form. (**f**–**i**) The delivery mechanism of AP-EGFP and TAT-EGFP. Jurkat T cells were pre-incubated with (**f**,**g**) MβCD or (**h**,**i**) heparin for 30 min before protein treatment. Error bars indicate SD * *p* ≤ 0.05; ** *p* ≤ 0.01; *** *p* ≤ 0.001; **** *p* ≤ 0.0001, ns = non-significant.

To identify the optimal sequence of AP for drug delivery to T cells, we generated several variants conjugated to EGFP by the deletion of arginine from both the N- and C-terminals of the original AP sequence: AP, RRRWCKRRR, AP(ΔR1), RRWCKRRR; AP(ΔR9), RRRWCKRR; AP(ΔR1ΔR9), and RRWCKRR. We incubated Jurkat T cells with 5 μM of each protein for 1 h and analyzed the intracellular delivery efficiency by flow cytometry. Deletion of a single arginine at either the N-terminal (AP(ΔR1)), C-terminal (AP(ΔR9)), or both (AP(ΔR1ΔR9)), significantly decreased intracellular delivery efficiency compared to the original AP sequence (Figure 2a,b), suggesting that three arginine residues at both ends are optimal for cargo delivery.

Tandem repeated forms of CPP increase delivery efficiency. We expected that the delivery efficiency could be enhanced by tandem repeats and generated tandem repeated forms of AP, dAP, and conjugated EGFP. The transduction efficiency of dAP-EGFP increased significantly compared to that of AP-EGFP at each dose (Figure 2c,d), but was not twice the threshold we set (Figure 2e). This result demonstrates that tandem repetition may not have a simple positive effect on delivery efficiency with an increased number of positively charged amino acids. As a result, we used the original AP as the optimal sequence for cargo delivery to T cells.

We hypothesized that the AP-conjugated cargo would mainly be delivered through endocytosis because of its size compared to other conventional CPPs. To investigate the mechanism of AP-mediated intracellular transduction in Jurkat T cells, we treated 5 µM of AP-EGFP or TAT-EGFP protein in Jurkat T cells, with heparin or methyl-β-cyclodextrin (MβCD) inhibitors. MβCD depletes cholesterol in lipid rafts and inhibits lipid raft-mediated endocytosis [21]. We used MβCD to identify lipid raft-mediated endocytosis because seven endocytic pathways, including phagocytosis and caveolae-mediated endocytosis, except for clathrin-mediated endocytosis, take place in lipid rafts [22]. Intracellular fluorescence decreased depending on the MβCD concentration and was strongly inhibited by heparin (Figure 2f–i), indicating that AP-EGFP was delivered into the cells through heparan sulfate-mediated interactions and utilized lipid raft-mediated endocytosis similar to TAT-EGFP.

### 3.3. The Cysteine Effect on the Transduction Efficiency to T Cells

**Figure 3:**
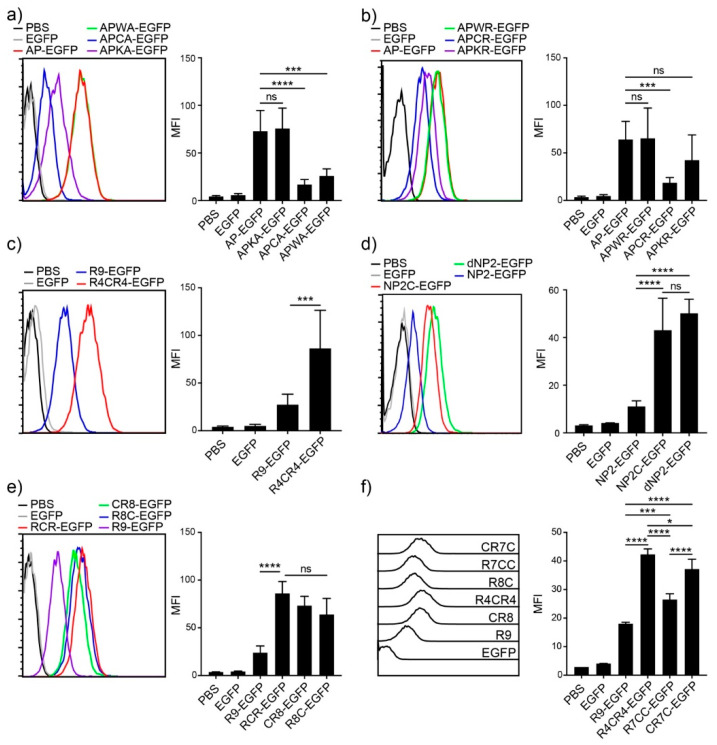
Cysteine effect on improving delivery efficiency (**a**–**f**) Comparison of the delivery efficiencies to Jurkat T cells. Jurkat T cells were incubated with 5 µM of CPP-EGFP proteins for 1 h. Intracellular fluorescence was analyzed by flow cytometry. (**left**) Histogram, (**right**) bar graph. Error bars indicate SD * *p* ≤ 0.05; *** *p* ≤ 0.001; **** *p* ≤ 0.0001, ns = non-significant.

Next, to investigate the key amino acid for the potent cellular transduction efficiency of AP, we generated mutant variants of AP by substituting tryptophan (W), cysteine (C), and lysine (K) with alanine (A) or arginine (R) in the middle of the AP sequence. When tryptophan, cysteine, and lysine residues were substituted with alanine or arginine, the lysine mutant exhibited reduced efficiency in alanine substitution, and the cysteine mutant showed a significant reduction in both cases, while the tryptophan mutant did not affect the intracellular transduction ability in either substitution case (Figure 3a,b), suggesting that cysteine is the critical amino acid for the intracellular protein delivery of AP.

Based on the results of increased efficiency by cysteine substitution, we hypothesized that adding cysteine residues to other CPPs would enhance their intracellular delivery efficiency. We first generated an R9 variant by substituting arginine with cysteine in the middle, R4CR4-EGFP, which showed more than three-fold efficiency compared to R9, as analyzed by flow cytometry (Figure 3c). We further generated the cysteine addition form of NP2, NP2c-EGFP, and compared it with the tandem repeat form of NP2, dNP2-EGFP. NP2c-EGFP exhibited approximately four-fold higher transduction efficiency than NP2-EGFP, and it was comparable to the tandem repeat form, dNP2-EGFP (Figure 3d). These results suggest that the simple addition of a single cysteine to CPPs can significantly enhance their transduction efficiency.

To determine whether the effect depends on the location of cysteine within the CPP sequence, we generated three different R9 variants with a single cysteine at the front, middle, or end of R9 (CR8, R4CR4, and R8C). There were no significant differences in the location of cysteine in the R9 sequence (Figure 3e). Next, we generated R9 variants with two cysteine residues to optimize the number of cysteine residues, CR7C-EGFP and R7CC-EGFP. The addition of two cysteines increased the delivery efficiency compared to R9, but a single cysteine addition was the most efficient (Figure 3f). In addition, the two cysteines at both ends of CPP showed more intracellular fluorescence intensity than the two serial cysteines. Collectively, these results suggest that cysteine is the key amino acid for enhancing delivery efficiency, and the addition of a single cysteine was optimal for delivery.

### 3.4. In Vivo Biodistribution and BBB-Penetration of AP

**Figure 4:**
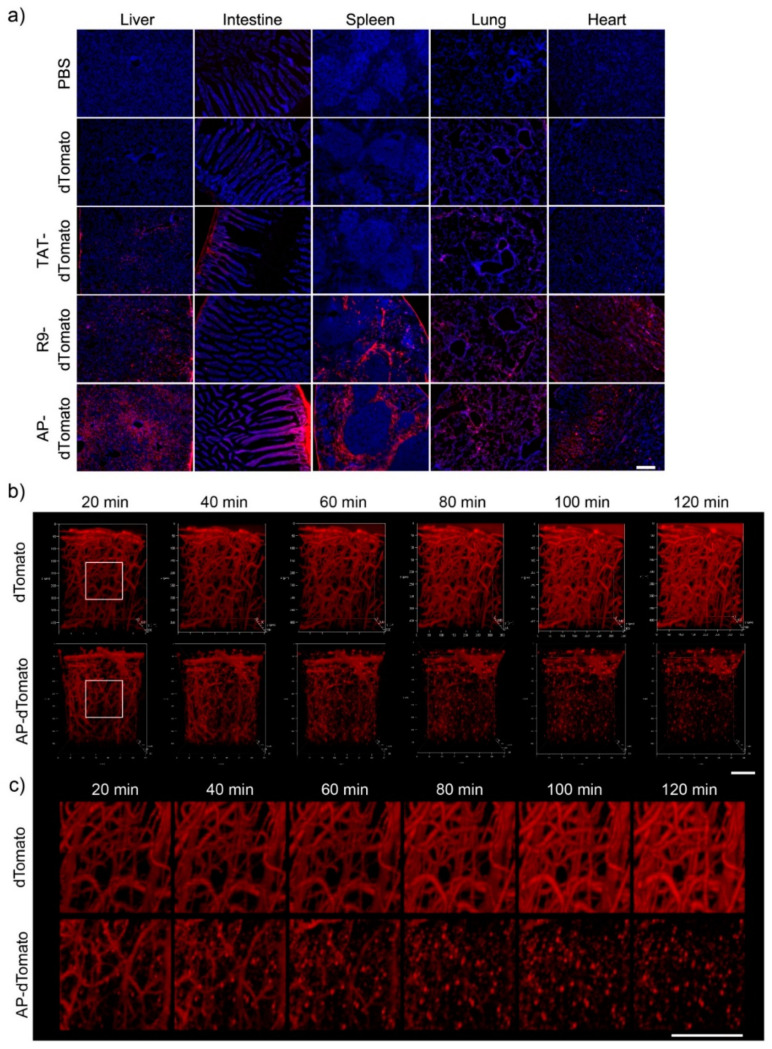
Biodistribution of CPP-dTomato proteins and the BBB-penetrating efficiency of AP (**a**) Biodistribution of CPP-dTomato proteins by intraperitoneal injection into mice. Overall, 5 mg of AP-dTomato and other control proteins were intraperitoneally administrated to C57BL/6 mice and the fluorescence in the tissues was analyzed 2 h after injection by fluorescence microscopy (×100, scale bar, 200 µm). (**b**,**c**) Real-time live confocal microscopic analysis of fluorescence in the brain. Overall, 2.5 mg of dTomato and AP-dTomato proteins were intravenously injected into eight-week-old female C57BL/6 mice. Mouse brains were observed 20 min after protein injection. The fluorescence in the brain tissue was visualized using a (**b**) 3D lateral view (scale bar, 50 µm) or (**c**) magnified view (scale bar, 50 µm).

To examine the *in vivo* feasibility of AP, we intraperitoneally injected 5 mg of AP-dTomato or other control proteins into C57BL/6 mice for 2 h and analyzed the fluorescence biodistribution of proteins in various organs, such as those of the liver, intestines, spleen, lungs, and heart, by fluorescence microscopy. Significant fluorescence of dTomato was detected in the liver, intestines, and spleen of AP-dTomato-treated mice compared to that of TAT-dTomato- or R9-dTomato-treated mice (Figure 4a). In a previous study, a novel BBB-permeable peptide, dNP2, significantly delivered dTomato protein across the BBB to brain tissue in a real-time *in vivo* multiphoton confocal imaging analysis, whereas TAT did not. Therefore, we performed real-time brain imaging after intravenous injection of 2.5 mg of dTomato and AP-dTomato proteins. AP-dTomato proteins were successfully visualized even at the early time point (20 min) after injection, and most of the proteins in the blood vessels had diffused out 100 min after injection (Figure 4b,c). In the case of dTomato, fluorescence was detected along the vasculature, and cellular structure was not observed up to 120 min after injection. These results suggest that AP can deliver cargo proteins to brain tissue with a much shorter peptide length than dNP2, indicating its potential for application in CNS inflammatory diseases.

### 3.5. T Cell Regulation in Th17 Differentiation and EAE by AP-ctCTLA-4

**Figure 5:**
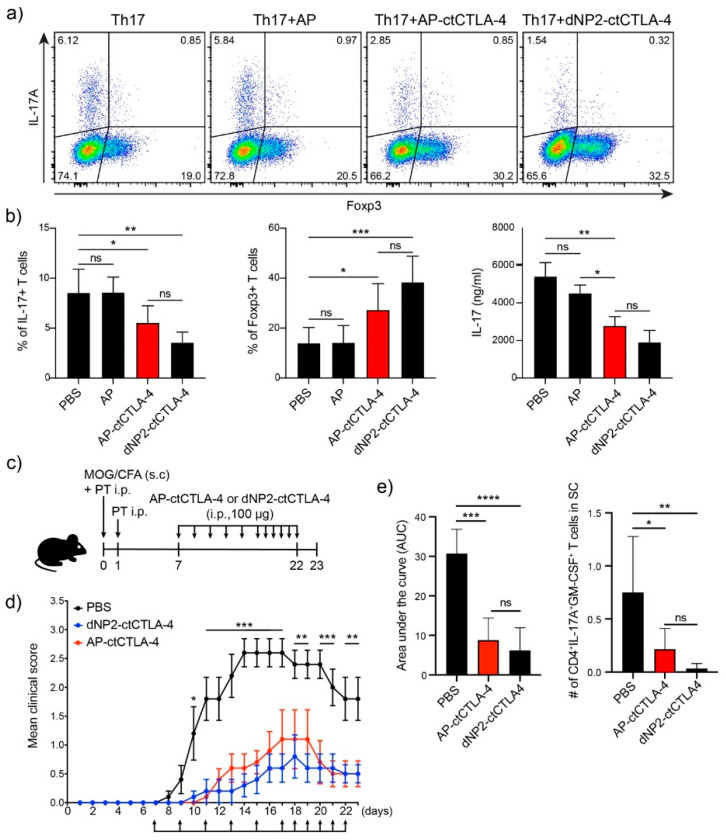
Regulation of Th17 cell differentiation and alleviation of EAE by AP-ctCTLA-4 (**a**) Regulation of Th17 cell differentiation by AP-ctCTLA-4. Mouse naïve CD4 T cells were stimulated with anti-CD3/CD28 mAb and cytokine cocktails with TGFβ for four days. Overall, 4 µM of peptides was treated on day 0. (**b**) Percentage of (left) IL-17 and (middle) Foxp3-expressing cells, and (right) secreted IL-17 level. (**c**) Experimental scheme of EAE induction. (**d**) Clinical score of EAE. (**e**) (left) area under the curve of the clinical score and (right) the number of infiltrated IL-17^+^GM-CSF^+^ pathogenic CD4 T cells in the spinal cord. Error bars indicate SD or SEM. * *p* ≤ 0.05; ** *p* ≤ 0.01; *** *p* ≤ 0.001; **** *p* ≤ 0.0001, ns = non-significant.

In our previous study, we found that dNP2-ctCTLA-4 could ameliorate the EAE clinical score by regulating T cells, by reducing IL-17 and inducing Foxp3 in an EAE mouse model [19]. We hypothesized that AP could exert an effect similar to that of dNP2 with a shorter peptide length. We first synthesized the AP-conjugated ctCTLA-4 peptide, which is 15 residues shorter than dNP2-ctCTLA-4. To determine whether AP-ctCTLA-4 could regulate Th17 differentiation *in vitro*, we sorted naïve CD4 T cells (CD4+CD25-CD62LhighCD44low) and differentiated them into Th17 cells with anti-CD3/28 antibodies and cytokine cocktails with TGF-β. *In vitro*, the AP-ctCTLA-4 peptide treatment in Th17 differentiation reduced IL-17+ cells and induced Foxp3+ T cells compared with dNP2-ctCTLA-4, whereas there were no effects on the AP peptide without the ctCTLA-4 treatment (Figure 5a,b). To confirm the therapeutic function of AP-ctCTLA-4 *in vivo*, we induced the EAE mouse model by immunizing with MOG35–55 peptide antigen on day 0 by subcutaneous injection. Then, we administered 100 µg of AP-ctCLTA-4 or dNP2-ctCTLA-4 peptides seven days after the immunization by intraperitoneal injection (Figure 5c). The clinical score and area under the curve were significantly reduced in the AP-ctCTLA-4-treated group of mice, with a significant decrease in pathogenic IL-17A^+^GM-CSF^+^ CD4 T cells in the spinal cord (Figure 5d,e). These results collectively suggest that AP-ctCTLA-4 peptide treatment also has a significant therapeutic effect on EAE disease progression by regulating T cells *in vivo*, with an even shorter CPP peptide length than dNP2.

### 3.6. Immunogenicity and In Vivo Single Dose Toxicity of AP-ctCTLA-4

**Figure 6:**
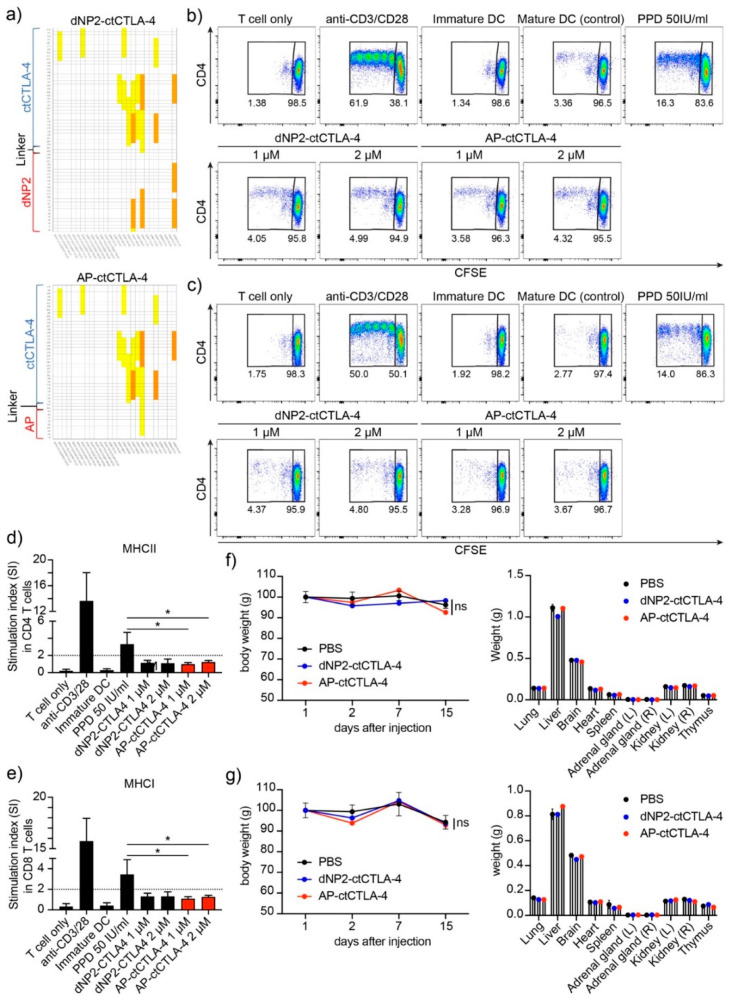
Evaluation of the immunogenicity and toxicity of AP-ctCTLA-4 compared with dNP2-ctCTLA-4 (**a**) *In silico* immunogenicity prediction of AP-ctCTLA-4 and dNP2-ctCTLA-4. (**b**–**e**) *In vitro* immunogenicity of peptides in hPBMC. Activation of (**b**,**d**) CD4 and (**c**,**e**) CD8 T cells cocultured with dendritic cells stained with CFSE and analyzed by flow cytometry. Single dose *in vivo* toxicity was analyzed in both (**f**) male and (**g**) female mice. Overall, 5 mg/kg of the peptides was injected intraperitoneally. Error bars indicate SD. * *p* ≤ 0.05.

To identify the potential of AP-ctCTLA-4 as a therapeutic agent, its immunogenicity and toxicity were evaluated. We first performed *in silico* immunogenicity prediction for the 27 most frequent HLA types. *In silico* immunogenicity prediction in dNP2 of dNP2-ctCTLA-4 showed a medium level of predicted immunogenicity in the three HLA types. In contrast, AP was expected to have a low level of immunogenicity in only one HLA type (Figure 6a). We further investigated the *in vitro* immunogenicity of one HLA-type blood sample. *In vitro* immunogenicity analysis using hPBMCs showed that both AP-ctCTLA-4 and dNP2-ctCTLA4 had no immunogenicity, with a stimulation index of less than two (Figure 6b–e), indicating that in terms of drug development, AP could be better applied in various human patients than dNP2.

In addition, to identify the *in vivo* toxicity of AP-ctCTLA-4, we injected 5 mg/kg, which is a 10-times greater dose of the effective dose in the EAE mouse model, and observed it for two weeks. In both the AP-ctCTLA-4 and dNP2-ctCTLA-4 peptides, there were no significant differences in body and organ weights after the single-dose injection (Figure 6f,g). These results suggest that both AP-ctCTLA-4 and dNP2-ctCTLA-4 can be developed as peptide drugs for multiple sclerosis, but AP might, in addition to the short peptide length, have another advantage in terms of immunogenicity while having a similar efficiency to dNP2.

## 4. Discussion

In the present study, we describe the feasibility of T cell regulation by AP, which was identified as a novel transdermal delivery peptide. We have evidenced the potent delivery efficiency of the AP peptide in both primary human T cells and Jurkat T cells. The cysteine residue in the middle of the AP sequence was found to be the residue critical for enhancing transduction efficiency to T cells, and this strategy could also be applied to other CPPs. In addition, we performed *in vivo* live imaging to visualize AP-mediated protein delivery to the brain across the BBB, which is generally regarded as a great hurdle for drug delivery. Moreover, we synthesized an AP-ctCTLA-4 peptide to regulate T cell function in an EAE mouse model, and it was observed that AP-ctCTLA-4 could regulate disease symptoms and significantly reduce pathogenic T cells compared with dNP2-ctCTLA-4.

T cells play a key role in the development or progression of diseases, such as autoimmune diseases, so controlling it is an important strategy for the prevention and treatment of diseases [23]. For this reason, many biological agents have been developed to modulate T cells, but are still confined to extracellular targets, and about 64% of human proteins are located in the intracellular region; thus, they remain unexplored as potential drug targets [9,10,11]. There are numerous potential targets in cells, but delivering macromolecular drugs to cells is technically challenging because of the impermeable cellular membrane [24]. T cells, especially primary T cells, are more challenging because they are small, non-phagocytic, with a high nuclear–cytoplasmic ratio and low endocytic rate [25,26].

Since the concept of CPPs, which have the ability to facilitate cellular uptake, was first identified in 1988, many attempts have been made to deliver macromolecules into cells, but relatively few studies have been conducted to deliver them to T cells or primary cells [27,28,29,30]. In the development stage of a new CPP, most cases evaluate the delivery efficiency in cancer cell lines *in vitro*, but their efficiency in primary cells is often low, which limits their clinical application [31]. One strategy is simply lengthening the number of basic amino acids, such as arginine or lysine. As shown in oligoarginine, transduction efficiency increases with increasing arginine counts up to R15 [32,33]. Similarly, tandem-repeat forms of CPP, such as dNP2 [18], 2pIL-1αNLS [34], d-Iduna [35], HHph-1 [36], and Pas2r12 [37], have improved delivery efficiency over single forms. Interestingly, cellular uptake was significantly improved by simply repeating the CPP sequence, but the degree differed greatly depending on the sequence (Figure 2e) or cell lines [35]. In the case of AP, the tandem repeat form of AP, dAP, showed improved delivery efficiency, but not as much as twice the cutoff value. Therefore, we proceeded with further experiments using the original sequence of AP, which is only a nine-amino acid peptide.

Although many studies of disease treatment have been conducted in animal models through cargo delivery using CPP, there are still hurdles in delivering drugs to tissues with barriers, such as the skin, intestines, or CNS [38]. In particular, the presence of the BBB which restricts transport to the CNS, renders the brain and spinal cord some of the most difficult targets for drug delivery [39]. Although some experimental results have evidenced the delivery of cargo molecules to the brain using CPPs, such as TAT, most of this transduction ability has been confirmed using indirect methods, such as β-gal analysis [40]. In contrast, we previously demonstrated by direct live imaging with multiphoton confocal microscopy, that a 24-mer of dNP2, a novel BBB-permeable peptide, can deliver proteins across the BBB to brain tissue [18]. In this study, we utilized a 9-mer AP peptide, which was developed as a novel transdermal delivery peptide, and found that AP exhibited potent protein delivery efficiency to brain tissue with a much shorter length of residues than dNP2. Moreover, by conjugating ctCTLA-4, AP successfully regulated Th17 cell differentiation of primary naïve CD4 T cells *in vitro* and ameliorated the clinical score in an EAE mouse model, comparable with dNP2-ctCTLA-4.

In terms of peptide drug development, the shorter the peptide length, the cheaper it is, making shorter peptides more competitive to market [41]. The AP peptide was only nine residues in length, significantly shorter than the 24-mer dNP2 peptide, and even AP delivered CTLA-4 to the cells, showing similar therapeutic effects to dNP2-ctCTLA-4 in the EAE mouse model. In addition, immunogenicity and toxicity were the first major evaluation items to be identified. Although both AP-ctCTLA-4 and dNP2-ctCTLA-4 showed no *in vitro* immunogenicity and *in vivo* toxicity, there was less predicted immunogenicity in the *in silico* analysis of AP-ctCTLA-4. Therefore, AP could have an advantage over dNP2 in terms of its length and potential immunogenicity.

In summary, we have demonstrated the feasibility of a transdermal delivery peptide, AP, in delivering cargo molecules to T cells and treating autoimmune encephalomyelitis by regulating T cells. This AP peptide with a single cysteine residue showed strong transduction efficiency in T cells, and the strategy of adding a single cysteine could be applied to improve the delivery efficiency compared to other CPPs. AP is a cell-penetrating peptide with a short length, and is expected to have comparatively low immunogenicity and potent macromolecule delivery efficiency to the brain; therefore, further research will focus on treating neurodegenerative diseases or Parkinson’s disease with AP-based drug delivery systems.

## 5. Conclusions

This study showed that successful delivery of cargo proteins across the BBB and disease control by regulating pathogenic CD4 T cells with the cytoplasmic domain of CTLA-4, was possible with the AP peptide upon systemic administration. This cysteine-containing AP peptide showed superior delivery efficacy in human T cells compared to TAT *in vitro*, and the effect of cysteine addition was also applicable to other CPPs. AP-conjugated proteins were transduced through heparan sulfate-and lipid raft-mediated endocytosis in T cells.

AP-conjugated proteins rapidly diffused out across the blood vessels in the brain within 20 min of systemic administration and accumulated in the brain tissue for at least 2 h, as analyzed by real-time multiphoton microscopy. This AP peptide could not only deliver proteins to the brain, but also control neuroinflammation in an EAE mouse model. The AP-conjugated cytoplasmic domain of CTLA-4 and AP-ctCTLA-4 inhibited Th17 differentiation *in vitro* and regulated inflammation by inhibiting pathogenic IL-17A^+^GM-CSF^+^ CD4 T cells *in vivo*. AP-ctCTLA-4 showed a low level of immunogenicity in *the in silico* analysis and no immunogenicity in one HLA type of hPBMC *in vitro*. In addition, it did not show *in vivo* toxicity in the dose range-finding toxicity experiment at 10-times the effective dose.

## Data Availability

Not applicable.
